# Comparative Study of the Rhizosphere and Root Endosphere Microbiomes of Cholistan Desert Plants

**DOI:** 10.3389/fmicb.2021.618742

**Published:** 2021-03-26

**Authors:** Salma Mukhtar, Samina Mehnaz, Kauser Abdulla Malik

**Affiliations:** KAM School of Life Sciences, Forman Christian College (A Chartered University), Lahore, Pakistan

**Keywords:** halophytes, root endosphere, Illumina sequencing, 16S rRNA gene, halophilic bacteria

## Abstract

Microbial communities associated with the rhizosphere and roots of desert halophytes play an important role in plants’ growth and development. Very limited information has been available on the microbial diversity of arid environments of Pakistan. Hence in the current study, the microbial diversity of rhizosphere and root endosphere of desert halophytes, *Zygophyllum simplex, Haloxylon salicoricum, Aerva javanica*, and *Capparis decidua* was evaluated. The rhizosphere and root endosphere samples of desert halophytes collected from the three geographic sites of Cholistan desert, Punjab, Pakistan were analyzed by using 16S rRNA based Illumina sequencing. The results showed that Proteobacteria were more abundant in the rhizospheric soils while Actinobacteria were more dominant in the root endosphere of halophytes. Bacteroidetes, Firmicutes, and Deinococcus-Thermus were identified from all rhizospheric soils and roots across the three sites, with variable percentage. *Bacillus, Kocuria, Pseudomonas, Halomonas*, and *Flavobacterium* were commonly identified from the rhizosphere and root endosphere of halophytes across all the three sites. At the genus level, microbial diversity from *Haloxylon* showed the greatest variations between the rhizosphere and root endosphere from the site 2. This study revealed that microbial diversity analysis can be used to study how changes in abiotic factors such as soil moisture content and salinity affect the microbial communities associated with the rhizospheric soils and root endosphere of halophytes across the three sites. This study will also help in the discovery of potential inoculants for crops growing in arid and semi-arid regions of Pakistan.

## Introduction

Soil salinity is considered as one of the major abiotic pressures that affects more than 840 million hectares of agricultural area worldwide. Soil salinity continuously increase in arid and semi-arid regions globally. Salinization reduces plant growth and adversely affects the crops yield and productivity (Rengasamy, 2006; Etesami and Beattie, 2018). Halophytes growing in arid and saline environments play a vital role in the maintenance of soil composition by nutrient mineralization and cycling, sequestration of carbon and improving the micro-environments (English and Colmer, 2011). These plants have great potential to preserve ecosystems. They can be used for production of biofuel and fiber and as fodder crops (Dagla and Shekhawat, 2005; Chaudhary et al., 2015).

Plant microbiome serves as a “second genome” to the plant and plays an important role in growth and productivity of halophytes. Rhizosphere and root endosphere microbiomes from extreme environments such as arid, saline, hot, cold, and acidic help the plants to grow in these environments (Oren, 2013; Santhanam et al., 2017; Mukhtar et al., 2018). Recent studies about rhizosphere microbiome of desert halophytes revealed a high portion of halophilic bacteria as compared to rhizosphere microbiome of salinity sensitive plants (Marasco et al., 2016; Tian and Zhang, 2017). A number of halophilic bacterial genera including *Bacillus, Halomonas, Halobacillus, Oceanobacillus, Marinobacter, Marinococcus*, and *Nesterenkonia* have been identified from the rhizosphere and roots of halophytes and xerophytes (Ramadoss et al., 2013; Zhao et al., 2016; Etesami and Maheshwari, 2018; Mukhtar et al., 2019).

It is well documented that halophilic and halotolerant PGPRs (plant growth promoting bacteria) enhance growth of halophytes (Yuan et al., 2016; Mukhtar et al., 2017a). These bacteria have the ability to improve plant growth and ultimately increase crop yield by nitrogen fixation, solubilization of minerals, production of phytohormones and siderophores, under salinity and drought conditions (Hussain et al., 2015; Zhou et al., 2017; Etesami and Beattie, 2018). PGPRs also provide plant protection against fungal and bacterial diseases by production of a variety of antifungal and antibacterial compounds (Bulgarelli et al., 2013). Halophiles are able to produce different industrially important enzymes such as protease, lipase, amylase, laccase, xylanase, and cellulase with polyextremophilic properties (Kumar et al., 2012; Mukhtar et al., 2019). Halophilic enzymes have potential for different industrial applications including textile, paper and pulp, pharmaceutical, baking, and detergent industries (Liszka et al., 2012; Mukhtar et al., 2019). Halophilic bacteria can also be used for bioremediation of a variety of hazardous compounds in saline and arid environments (Liszka et al., 2012; Castillo-Carvajal et al., 2014; Tian and Gao, 2014).

A number of studies have already reported desert halophytes and xerophytes, such as Cactus, Agave, *Halocnemum, Halostachys*, *Lycium, Salicornia*, and *Kalidium* associated microbial diversity by using high throughput sequencing approaches (Coleman-Derr et al., 2016; Fonseca-Garcia et al., 2016; Yuan et al., 2016; Tian and Zhang, 2017). However, the microbial communities associated with the desert halophytes growing in Cholistan, Pakistan, an extremely dry and saline environment, have not been previously explored. Authors evaluated the rhizosphere and root endosphere microbiomes of four desert halophytes *Zygophyllum simplex, Haloxylon salicoricum, Aerva javanica*, and *Capparis decidua*, collected from three sites by using culture-independent (Illumina sequencing) approaches. The main objectives of the present study were: (1) to compare the microbial communities associated with the rhizosphere and root endosphere of each halophyte, individually, among the three geographic sites and (2) to compare the microbial diversity from the rhizosphere and root endosphere of four halophyte species so as to understand the differences among the plant species and across the three geographic sites.

## Materials and Methods

### Soil Sampling

Cholistan is a hot arid sandy desert covering an area of 26,000 km^2^ and is locally known as Rohi. It is situated in the South-West of Punjab province (Pakistan) and has an average annual rainfall from 128 to 178 mm (Supplementary Figure 1). Geographically, it is located 27°42′ and 29°45′ North, 69°52′ and 75°24′ East. Water is available at 25–90 m depth and is too brackish. Drought in this region is quite common, sometimes extending from 2 to 3 years, causing a lot of harms (Chaudhry and Nasim, 1995). Vegetation in this area includes a variety of grasses (*Panicum, Cenchrus, Aristida*, and *Lasiurus*), herbs (*Suaeda, Chenopodium*, *Aerva, Zygophyllum*, and *Dipterygium*), and shrubs (*Haloxylon*, *Justicia, Capparis*). Authors have surveyed an area of approximately 71 km near the Lal Suhanra National Park, Cholistan, and selected three geographic sites (approximately 23 km far from each other) for sampling of halophytes according to land use and vegetation cover (Supplementary Figure 2). These sampling sites were considered as the most drought affected regions. Drought tolerant halophytes, including *Zygophyllum simplex, Haloxylon salicoricum, Aerva javanica*, and *Capparis decidua*, dominant across all three sites, were collected. The sampling area was selected according to land use and vegetation cover. The whole plants were excavated with surrounding soil in blocks (15–20 cm in depth, 20 cm in width, and 20 cm in length). Four replicates for each plant and soil sample were collected from each site (Koranda et al., 2011; Mukhtar et al., 2018). Plant and soil samples were taken to the laboratory from the collection site in an ice box and stored at −80°C for microbial diversity analysis.

### Soil Physicochemical Characteristics

About 350 grams of soil were dried and sieved for estimation of physicochemical properties. The pH of soil was measured by preparing a mixture of 1:2.5 (w/v) soil to water. Electrical conductivity (dS/m) was calculated according to Adviento-Borbe et al. (2006). Soil moisture and texture class were estimated by using the method from Anderson and Ingram (1993). Total organic carbon (C_org_) was determined by Walkley and Black (1934) method (1934) and total organic nitrogen was calculated using Kjeldahl method. Available phosphorous (P) was estimated by using a calorimetric method with sodium bicarbonate, ammonium molybdate, and ascorbic acid (Olsen et al., 1954). Potassium (K), calcium (Ca), and magnesium (Mg) were detected by atomic absorption spectrometry. Carbonate (CO_3_^2–^) and bicarbonate (HCO_3_^–^) ions were determined by using Vogel (1978) method (1978).

### DNA Extraction, Amplification of 16S rRNA Gene and Illumina MiSeq Sequencing

About 100 g of rhizospheric soil was mixed thoroughly and filtered with 2 mm sieve. The total DNA from soil samples (0.5 g) were extracted using FastDNA Spin Kit for Soil, according to manufacturer’s instructions. For DNA extraction from root endosphere, roots were surface sterilized according to Mukhtar et al. (2019) and FastDNA Spin Kit specific for plant tissues was used. The quality of DNA was determined by using 0.9% agarose gel and quantity was measured by using Nanodrop (NanoDrop 200c Thermo Fisher Scientific, Waltham, MA, United States).

In total, 96 DNA samples; 12 rhizosphere and 12 root samples of each plant collected from the three geographic sites were used for amplification of 16S rRNA gene and Illumina (MiSeq) sequencing. The V3–V4 region of the 16S rRNA gene was amplified by using primers (Bakt_341F: CCTACGGGNGGCWGCAG and Bakt_805R: GACTACHVGGGTATCTAATCC), which were linked with unique identifier and adapter sequences (Supplementary Table 1). The detailed PCR conditions for amplicon sequencing were the same as described by Herlemann et al. (2011). Amplified PCR products were purified with Agencourt AMPure beads (Beckman Coulter, Brea, CA, United States). Finally, about 10 ng of DNA from each sample was sequenced on the Illumina MiSeq platform by Macrogen (Geumcheon-gu, Seoul, South Korea).

### Bioinformatics and Statistical Analyses

Sequences were processed and sorted using the default parameters in QIIME 1.3 (Caporaso et al., 2010). An offset of 10 nucleotides was set in order to remove the first 10 bases of each sequence, and high quality sequences with an average length of 350 bases were selected. Chimera Slayer software was used to check chimeric sequences (DeSantis et al., 2006). *De novo* OTU (operational taxonomic unit) picking was done to generate OTU files using UCLUST that is a default parameter of QIIME, with 97% sequence similarity and RDP classifier was used to assign taxonomy (Wang et al., 2007). For different taxonomic levels, such as phylum, class, order, family, and genus, Good’s coverage was calculated by using 97% similarity cutoff (Good, 1953).

Alpha and beta diversity were determined using QIIME alpha_rerefaction.py and beta_diversity_through_plots.py commands, respectively. Alpha diversity was calculated at a sequence depth of 82,152 reads per soil sample, as alpha diversity indices are correlated with the number of sequences by using the Kruskal–Wallis test. The selected maximum sampling depth corresponded to minimum number of reads obtained from any of the remaining sequenced samples. Beta diversity was analyzed by using non-metric multidimensional scaling analysis (NMDS) in “Mass” and “vegan” packages. A matrix was calculated using the weighted and unweighted UniFrac distances among samples at a sequence depth of 82,152 reads per soil sample (Lozupone et al., 2011). Distances were calculated using the “envfit” function of the package “vegan” for Bray-Curtis. To further explore the relationship between bacterial communities and soil properties, redundancy analysis (RDA) and mental test were performed to study the relationship between the most abundant bacteria and soil properties. To explain the differences in the composition of taxa inside the data matrix community, a heatmap (relative abundance matrix) was generated at class level using XLSTAT 7.0 software (Fahmy, 2003). Number of OTUs was calculated by using Venn diagrams analysis and distances were calculated using the “vegdist” function of the “vegan R package.” Variation partitioning was performed on the basis of plant species, soil physicochemical characteristics, and geographical distance on composition of rhizosphere and root endosphere microbiomes across the three sites.

To detect the taxonomic classifications that were significantly abundant in rhizospheric soils and root samples, Wilkcoxon’s non-parametric rank-sum test and LDA using the LEfSe (LDA Effective Size) program was used (Segata et al., 2011). One-way ANOVA was applied to analyze differences among rhizospheric soils and root samples. Environmental fitting analysis using “envfit” function in vegan R package was used to find out which soil physicochemical factors strongly associated with rhizosphere and root endosphere microbiomes. To predict the functional profile of bacteria identified from the rhizosphere and root endosphere of halophytes, Tax4Fun software (R package) was used (Abhauer et al., 2015). Variation partitioning analysis was performed to study the impact of the relative influences of plant species, soil physicochemical characteristics, and geographical distance on composition of rhizosphere and root endosphere microbiomes. Retrieved 16S rRNA sequences datasets were deposited in NCBI GenBank under SRA accession numbers SRR9588854 to SRR9588861.

## Results

### Correlations Between Physicochemical Characteristics of Soil and Microbial Community Structure

Soil pH ranged from 7.02 to 7.69 with the maximum value in *Haloxylon* soils collected from the site 2 and the minimum in *Zygophyllum* soils collected from the site (Table 1), electrical conductance (EC_1__:__1_) ranged from 3.79 to 5.51 dS/m with the maximum value in *Haloxylon* soils collected from the site 2 and minimum in *Aerva* soils collected from the site 3 (Table 1). The moisture content ranged from 20.25 to 25.49%. The soil samples collected from site 2 were more dried as compared to soil samples from the geographic sites 1 and 3. Soil temperature ranged from 38.79 to 42.54°C (Table 1). The average organic matter ranged from 12.55 to 18.37 g.kg^–1^ with the highest values in *Capparis* rhizospheric soils collected from site 2 and the lowest in *Haloxylon* rhizospheric soils collected from site 1. The average values for P, K, Ca, and Mg contents were 6.78, 0.55, 182.25, and 82.16 mg.kg^–1^, respectively. The values of nitrate ions were higher in *Capparis* rhizospheric soils as compared to other plants soils from all sites. Carbonate and bicarbonate ions were higher in *Haloxylon* and *Aerva* rhizospheric soils, as compared to other plants soils (Table 1).

**TABLE 1:** Physical and chemical properties of rhizospheric soil samples of desert halophytes.

**Soil properties**	**Site 1**				**Site 2**				**Site 3**			
	***Zygophyllum***	***Haloxylon***	***Aerva***	***Capparis***	***Zygophyllum***	***Haloxylon***	***Aerva***	***Capparis***	***Zygophyllum***	***Haloxylon***	***Aerva***	***Capparis***
**pH**	7.02^a^	7.26^a^	7.21^a^	7.51^b^	7.53^b^	7.69^a^	7.29^a^	7.11^a^	7.11^a^	7.57^b^	7.19^a^	7.47^b^
**EC_1__:__1_ (dS/m)**	4.14^a^	4.77^b^	3.85^a^	4.17^ab^	4.75^b^	5.51^c^	4.59^b^	4.21^ab^	3.79^a^	4.27^ab^	3.59^a^	4.47^b^
**Moisture (%)**	22.15^a^	20.78^a^	22.23^a^	25.49^b^	24.34^ab^	20.25^ab^	21.57^a^	23.55^a^	23.24^a^	21.26^a^	22.67^a^	25.29^b^
**Temperature (°C)**	40.21^a^	42.54^b^	41.71^ab^	41.83^ab^	40.87^ab^	41.11^ab^	41.49^b^	40.98^ab^	38.79^a^	39.15^a^	39.27^a^	39.61^a^
**Texture class**	Sandy soil	Sandy soil	Sandy soil	Sandy soil	Sandy loam	Sandy loam	Sandy loam	Sandy loam	Sandy loam	Sandy loam	Sandy loam	Sandy loam
**OM (g.Kg**^–^**^1^)**	16.31^ab^	12.55^a^	15.61^ab^	17.37^b^	15.53^ab^	17.61^b^	14.89^ab^	18.35^b^	16.49^ab^	11.71^a^	16.53^ab^	17.91^b^
**P (mg.kg**^–^**^1^)**	7.05^ab^	7.66^b^	6.29^a^	6.14^a^	6.97^ab^	7.69^b^	6.45^a^	6.11^a^	7.77^b^	6.81^ab^	6.19^a^	6.33^a^
**K (mg.kg**^–^**^1^)**	0.47^a^	0.51^a^	0.59^b^	0.65^b^	0.44^a^	0.59^b^	0.45^a^	0.61^b^	0.49^a^	0.63^b^	0.61^b^	0.60^b^
**Ca (mg.kg**^–^**^1^)**	131.41^a^	187.45^b^	205.49^b^	179.32^b^	157.11^a^	207.53^b^	211.12^b^	191.49^b^	149.21^a^	199.57^b^	209.55^b^	157.73^a^
**Mg (mg.kg**^–^**^1^)**	64.47^a^	97.05^b^	71.22^a^	103.4^b^	67.73^a^	89.29^ab^	93.13^ab^	69.37^a^	65.47^a^	91.44^ab^	109.52^b^	93.67^ab^
**NO_3_**^–^ **(mg.kg**^–^**^1^)**	9.56^a^	10.13^a^	9.17^a^	12.97^b^	10.09^a^	10.33^a^	9.99^a^	13.25^b^	9.97^a^	10.17^a^	10.37^a^	12.65^b^
**CO_3_^2^**^–^ **(mg.kg**^–^**^1^)**	17.56^a^	24.29^b^	22.19^b^	15.53^a^	18.19^a^	23.53^b^	19.93^ab^	17.79^a^	18.56^a^	23.21^b^	22.78^b^	19.17^a^
**HCO_3_**^–^ **(mg.kg**^–^**^1^)**	1.51^a^	2.61^b^	2.75^b^	1.93^ab^	1.93^ab^	2.77^b^	2.45^b^	1.49^a^	1.59^a^	2.59^b^	2.67^b^	1.95^ab^

*EC, Electrical conductivity; OM, Organic matter; P, Phosphorous; K, Potassium; Ca, Calcium; Mg, Magnesium; NO_3_^–^, Nitrate; CO_3_^2–^, Carbonate ion; HCO_3_^–^, Bicarbonate ion; Letters represent statistically different values at 5% level.*

**FIGURE 1:**
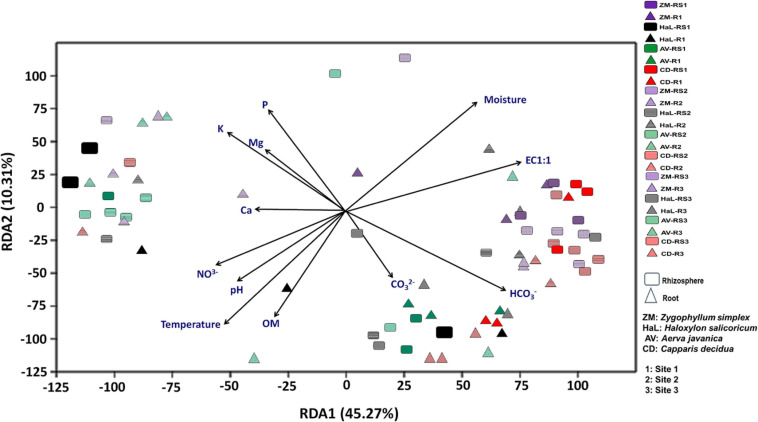
Environmental fitting analysis of rhizosphere and root endosphere microbiomes across the three sites by using redundancy analysis (RDA) plot.

Differences in community structure among different rhizospheric soil and root samples across the three geographic sites were explained by redundancy analysis (RDA) (Figure 1). It was observed that microbial communities in the rhizosphere and roots of *Haloxylon* and *Zygophyllum* showed greatest variations as compared to microbial communities identified from the rhizosphere and roots of *Aerva* and *Capparis*, from the geographic site 2 (Figure 1). Some bacterial genera were more abundant than others at each sampling site. Soil physicochemical characteristics, especially salinity and moisture content affect the microbial diversity in each plant across the three sites.

### General Characteristics of 16S rRNA Based Illumina Sequencing

In this study, a total of 697743 sequences were obtained from the rhizospheric soil samples and 647802 sequences from the root endosphere of halophytes, before the removal of mitochondrial and plastid contaminants while 638292 sequences were obtained from the rhizospheric soil samples and 530006 sequences from the root endosphere of halophytes, after the removal of mitochondrial and plastid contaminants (Supplementary Table 2).

About 67–83% reads were assigned at the phylum level, 59–81% to class level, 34–51% to the family, and 31–49% were assigned to the genus level, from the geographic site 1 (Supplementary Figure 3). About 71–81% reads were assigned at the phylum level, 51–72% to the class level, 34–51% to the family, and 27–50% were assigned to the genus level, from the geographic site 2 (Supplementary Figure 4). About 69–81% reads were assigned at the phylum level, 57–71% to the class level, 29–55% to the family, and 26–45% were assigned to the genus level from the geographic site 3 (Supplementary Figure 5).

### Microbial Diversity Comparisons at Global OTU Level

**FIGURE 2:**
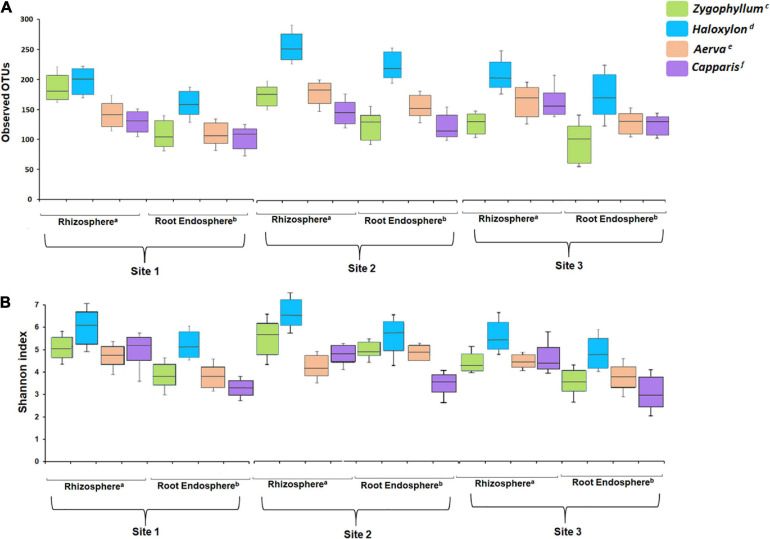
Alpha diversity analysis. **(A)** Number of observed OTUs and **(B)** Shannon index. All the soil and root samples showed significant difference after a Kruskal–Wallis test with a confidence level of 99% (*p* ≤ 0.05) in each index. Superscripts (a and b) indicate significant differences in the rhizosphere and root endosphere among plant species, while superscripts (c–f) indicate significant differences between sample types associated with a plant species.

The results for observed OTUs for microbial communities identified from the rhizosphere and root endosphere of *Haloxylon* were highly variable as compared to soil and root samples of other plants, collected across all sites (Figure 2A and Supplementary Table 3). Within plant species, alpha diversity was highest in the rhizosphere and root endosphere of *Haloxylon* from the geographic site 2. Overall, microbial communities from the rhizosphere of all plants showed higher diversity as compared to the root endosphere and microbial diversity differed significantly among plant species and across the three sites (Figure 2B and Supplementary Table 4).

**FIGURE 3:**
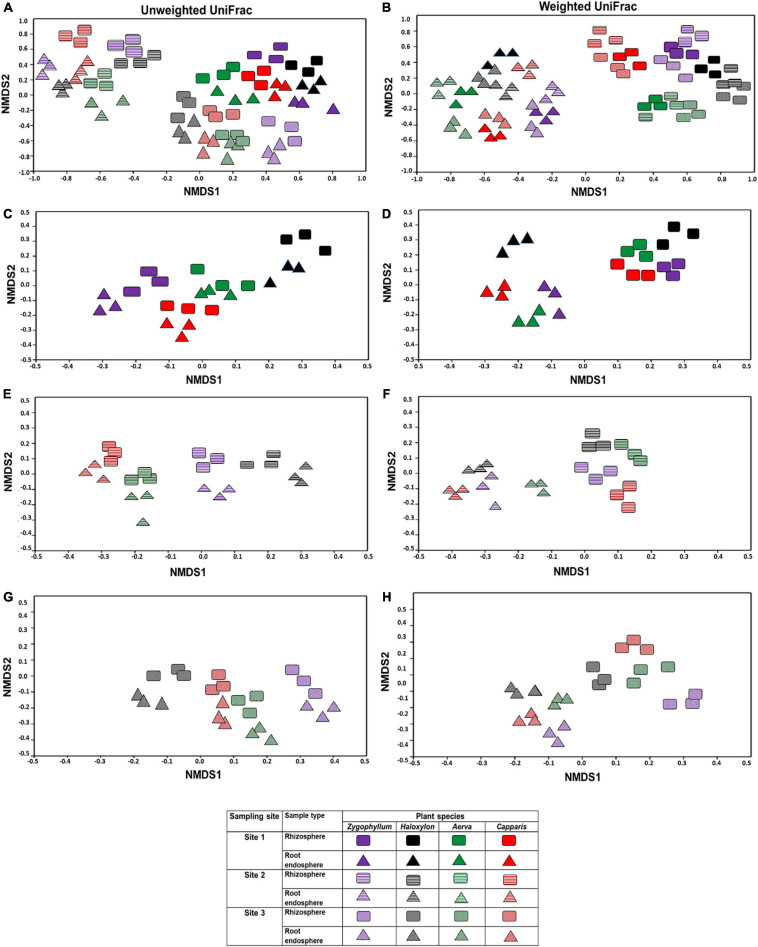
Beta diversity index of the bacterial communities in the rhizosphere and root endosphere of desert halophytes. Beta diversity was calculated by using weighted and unweighted UniFrac distances for non-metric multidimensional scaling plots **(A,B)** samples collected from all the sites, **(C,D)** samples collected from only site 1, **(E,F)** samples collected from only site 2, and **(G,H)** samples collected from only site 3 using unweighted **(A,C,E,G)** and weighted **(B,D,F,H)** UniFrac distances.

Beta diversity was calculated by using weighted and unweighted UniFrac distances for non-metric multidimensional scaling plots. Microbial communities associated with the rhizosphere and root endosphere were clustered significantly different among the plant species and across the sites (Figures 3A,B). In case of site 1, microbial diversity from the rhizosphere samples was clustered differently from the root endosphere samples, within and between plant species (Figures 3C,D). The microbial communities identified from the site 2 showed higher variations among plant-type and sample-type as compared to microbial communities identified from the site 1 and site 3 (Figures 3E–H, respectively). Overall, the NMDS analysis showed that the rhizosphere and root endosphere microbiomes of *Haloxylon* exhibited the highest variations from the site 2.

### Microbial Diversity Comparisons at the Phylum and Class Level

**FIGURE 4:**
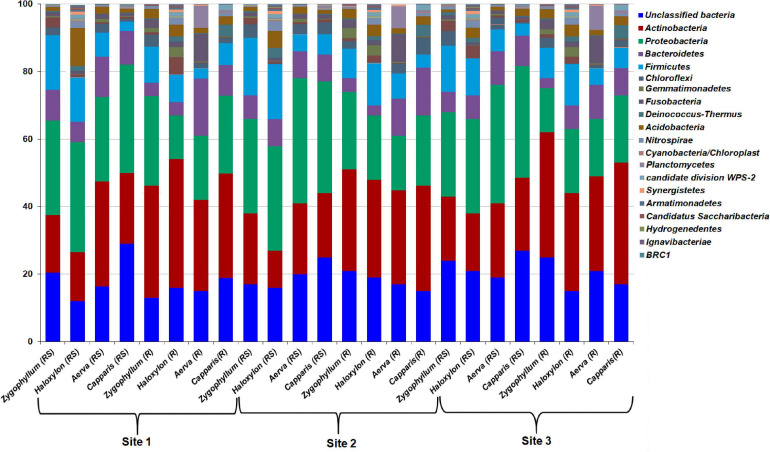
Relative abundance of bacterial phyla from the rhizosphere (RS) and root endosphere (R) of desert halophytes collected from three sites of Cholistan.

The OTUs from all the soil and root samples were assigned to 19 bacterial phyla. Microbial diversity at the phylum level showed significant differences among rhizosphere and root endosphere of halophytes across the three geographic sites (Figure 4). *Proteobacteria* (25.31–37.13%) was the most abundant bacterial phylum identified from the rhizospheric soil samples of all plants while *Actinobacteria* (27.23–38.07%) were dominant in root endosphere of all plants, across the three sites. Members of *Firmicutes* (8.51–17.19%) were dominant in the rhizosphere and roots of *Zygophyllum* and *Haloxylon* whereas in case of *Aerva* and *Capparis, Bacteroidetes* (8.25–13.12%) showed abundance across the three sites. Sequences related to Chloroflexi, Gemmatimonadetes, Fusobacteria, Deinococcus-Thermus, Acidobacteria, and Nitrospirae, were relatively less abundant, however, detected from all the rhizospheric soils with a significant difference in abundance across the three sites (Figure 4). At the phylum level, microbial communities associated with the rhizosphere and root endosphere of *Haloxylon* showed the highest variations as compared to microbial communities, identified from the rhizosphere and root endosphere of other desert halophytes, across the site 2. Our results also indicated that *Actinobacteria* significantly correlated with the roots and *Proteobacteria* significantly correlated with the rhizosphere of halophytes, whereas Nitrospirae and Synergistetes correlated least with the rhizosphere of halophytes, across the three sites.

The relative abundance of bacterial classes from the rhizosphere and roots of desert halophytes collected from the three geographic sites were compared in the form of a heatmap (Supplementary Figure 6). Bacteroidia, Alphaproteobacteria, Bacilli, Betaproteobacteria, Sphingobacteria, Deltaproteobacteria, Gammaproteobacteria, Planctomycetia, Clostridia, and Halobacteria, were found to be abundant classes identified from all the rhizosphere and root samples of halophytes, collected from the site 1 (Supplementary Figure 6A). Bacilli, Alphaproteobacteria, Betaproteobacteria, Bacteroidia, Deltaproteobacteria, Flavobacteriia, Planctomycetia, Sphingobacteria, Acidobacteria_Gp7, Acidobacteria_Gp9, Nitrospira, and Halobacteria were dominating classes detected from all the rhizosphere and root samples of halophytes, collected from the site 2 (Supplementary Figure 6B). Bacilli, Alphaproteobacteria, Bacteroidia, Gammaproteobacteria, Deltaproteobacteria, Flavobacteriia, Planctomycetia, Acidobacteria_Gp9, Chlamydiia, Sphingobacteria, Nitrososphaeria, Acidobacteria_Gp7, Deinococci, Holophagae, and Halobacteria were dominating classes detected from all the rhizosphere and root samples of halophytes, collected from the site 3 (Supplementary Figure 6C). The cluster structure showed a different distribution pattern in rhizospheric soils as compared to root endosphere of halophytes, across the three sites. At class level also, the highest variations were observed between the rhizosphere and root endosphere of *Haloxylon*, collected from the geographic site 2.

### Microbial Diversity Comparisons of Rhizosphere and Root Endosphere Across Three Geographic Sites at Genus Level

**FIGURE 5:**
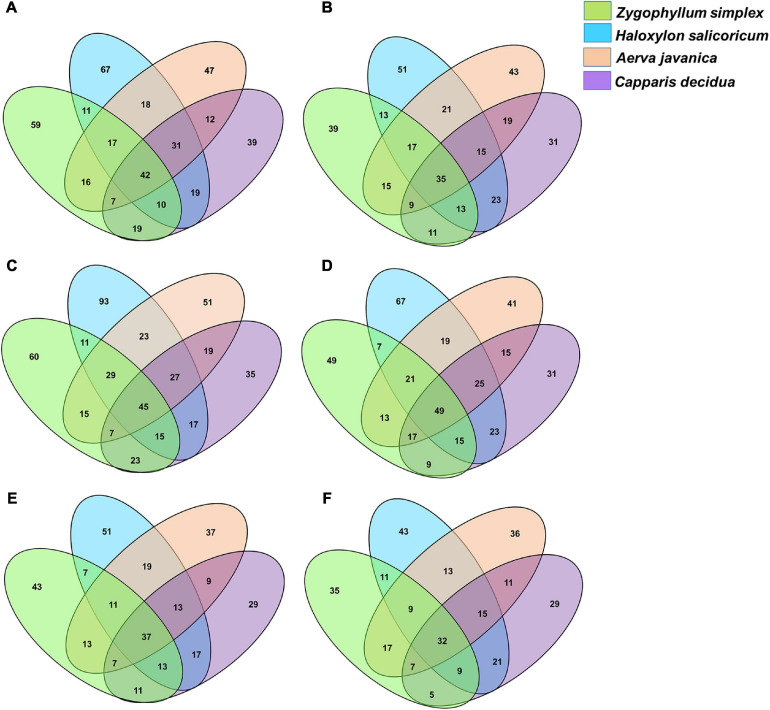
Venn diagram showing the numbers of bacterial genera identified from the rhizosphere and the root endosphere of desert halophytes. **(A,B)** Samples collected from the site 1, **(C,D)** Samples collected from the site 2, and **(E,F)** Samples collected from the site 3.

The phylotypes identified from the rhizosphere and root endosphere of halophytes were compared across the three geographic sites. Venn diagram analysis revealed that a total of 765 phylotypes from the rhizosphere and 682 phylotypes from the root endosphere of halophytes have been identified from the site 1 (Figures 5A,B), 869 phylotypes from the rhizosphere and 790 phylotypes from the root endosphere of halophytes from the site 2 (Figures 5C,D), and 592 phylotypes from the rhizosphere and 547 phylotypes from the root endosphere of halophytes have been identified from the site 3 (Figures 5E,F).

Overall, maximum microbial diversity was detected from the rhizosphere and root endosphere of all plants collected from the geographic site 2. A total of 45 phylotypes were commonly identified from all the rhizospheric soils and 60, 93, 51, and 35 phylotypes were exclusively identified from the rhizosphere of *Zygophyllum, Haloxylon, Aerva*, and *Capparis*, respectively (Figure 5C). From the root endosphere of halophytes, 49 phylotypes were commonly identified and 49, 67, 41, and 31 phylotypes were exclusive to the root endosphere associated microbial diversity of *Zygophyllum, Haloxylon, Aerva*, and *Capparis*, respectively (Figure 5D).

**FIGURE 6:**
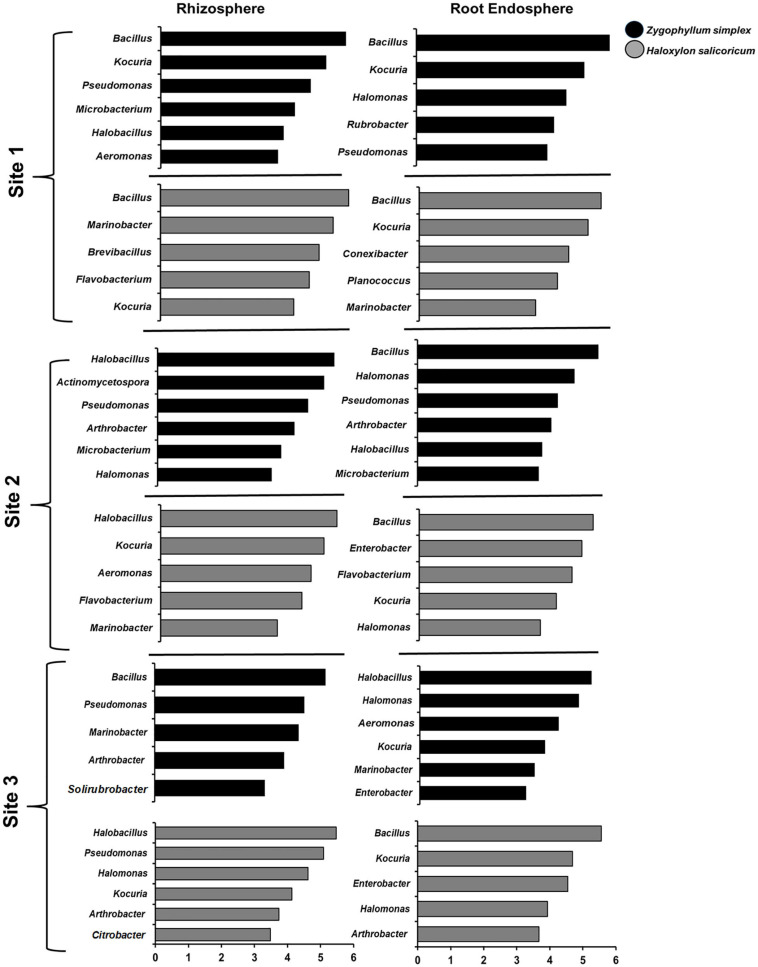
The results of LEfSe analysis showed the identified bacterial genera that were significantly abundant in the rhizosphere and root endosphere of halophytes. Variations in the bacterial genera detected across the rhizosphere and the root endosphere of *Zygophyllum* and *Haloxylon* from three sampling sites. Bacterial genera with the LDA score of more than 3.5 are shown.

The results of LEfSe analysis showed the differences among the dominant bacterial genera from the rhizosphere and root endosphere of halophytes across all the sampling sites (Figure 6 and Supplementary Figure 7). The top five bacterial genera based on LDA scores were compared among the sample-type, plant species and across the sites. Bacterial genera, including *Bacillus, Kocuria, Pseudomonas, Halomonas*, and *Flavobacterium* were commonly identified from the rhizosphere and root endosphere of halophytes across all the geographic sites (Figure 6, Supplementary Figure 7 and Supplementary Tables 5–7). All the plant species showed great variations from one plant to another, however, within sample-type (the rhizosphere and root endosphere), there was no significant variation. Bacterial genera *Virgibacillus, Oceanobacillus*, and *Planococcus* (Firmicutes); *Aeromonas, Marinobacter, Enterobacter*, and *Citrobacter* (Proteobacteria); *Kocuria, Solirubrobacter, Rubrobacter, Microbacterium*, and *Arthrobacter* (Actinobacteria); were exclusively identified from the rhizosphere and root endosphere of *Zygophyllum* and *Haloxylon* while *Burkholderia, Serratia*, and *Klebsiella* (Proteobacteria), and *Polaribacter* (Bacteroidetes) were identified only from the rhizosphere and root endosphere of *Aerva* and *Capparis*. At the genus level, *Haloxylon* showed the greatest variations between the rhizosphere and root endosphere from the site 2 (Figure 6 and Supplementary Table 6). These results also confirmed the abundance of bacterial phyla Proteobacteria, Actinobacteria, Firmicutes, and Bacteroidetes, as the top bacterial genera from all the soil and root samples across all sampling sites.

### Factors Influencing Bacterial Community Structure and Distribution

**FIGURE 7:**
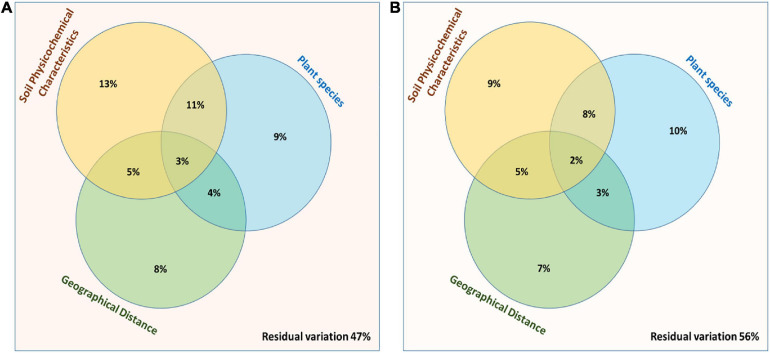
Partitioning between the biological variations in the bacterial community structure. Three explanatory matrices were used here, containing variables pertaining to plant species, soil physicochemical characteristics and geographical distance on composition of **(A)** Rhizosphere and **(B)** Root endosphere microbiomes.

A multivariate variation partitioning approach was used to study the effects of soil physicochemical characteristics and the geographical distance on the overall variation of rhizosphere and root endosphere microbiomes based on the most abundant OTUs. A combination of environmental parameters; plant species, soil physicochemical characteristics, and geographical distance could explain 53% of their overall biological variations while 47% variations were unexplained in case of rhizosphere microbiomes (Figure 7A) while from the root endosphere microbiomes, different factors could explain only 44% of their overall biological variations while 56% variations were unexplained (Figure 7B).

### Functional Profile of Bacterial Communities

**FIGURE 8:**
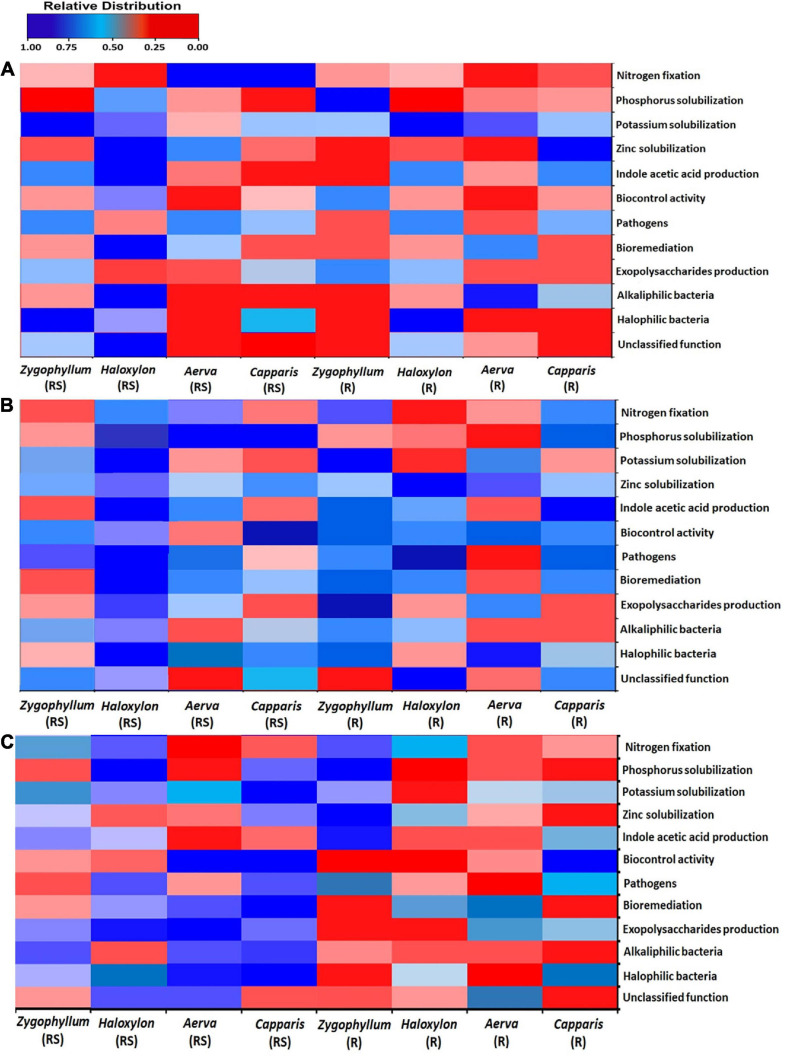
Bacterial linked to different functional categories in the rhizosphere (RS) and root endosphere (R) of desert halophytes collected from the three sites; **(A)** Site 1, **(B)** Site 2, and **(C)** Site 3.

The results for the prediction of functional profiles of bacterial genera with specific plant growth promoting traits showed that nitrogen fixers were more abundant in the root endosphere of all the halophytes across the geographic sites 1 and 2 (Figures 8A,B). Mineral (Phosphate, potassium, and zinc) solubilizers and IAA (indole-3-acetic acid) producers were more dominant in the rhizosphere of *Zygophyllum, Haloxylon*, and *Aerva* as compared to root endosphere across all sites. Bacterial strains with biocontrol activity were found to be most abundant in the rhizosphere of *Haloxylon* and *Capparis*, from the site 2 (Figures 8B,C). Microbes involved in bioremediation of toxic compounds were more abundant in the rhizosphere and root endosphere of *Haloxylon, Aerva*, and *Capparis*, across all sites. More than 83% bacterial strains identified in this study were halophilic and alkaliphilic in nature (Figures 8A,B).

## Discussion

Bacterial strains isolated from the extreme environments have special physiological and genetic characteristics to survive under such conditions. Cholistan desert is considered as naturally occurring extreme environment with drought, salinity and high temperature as the main abiotic factors. In this study, microbial diversity was compared from the rhizosphere and root endosphere of desert halophytes such as *Zygophyllum simplex, Haloxylon salicoricum, Aerva javanica*, and *Capparis decidua*, collected from three geographic sites of Cholistan desert, Pakistan by using 16S rRNA based Illumina sequencing. The main objective of this study was to evaluate the impact of plant species, sample-type (rhizospheric and root endosphere) and geographic site, on the composition of microbial communities associated with desert halophytes. A number of studies have been previously reported on microbial diversity analyses from halophytes and xerophytes, such as microbial communities associated with the root endosphere of *Halocnemum, Halostachys*, *Lycium*, and *Salicornia*, using high throughput sequencing approaches (Coleman-Derr et al., 2016; Yuan et al., 2016; Tian and Zhang, 2017; Fitzpatrick et al., 2018). Here, we have reported the microbial diversity associated with the rhizosphere and root endosphere of halophytes, collected from Cholistan, Pakistan, by using culture-independent approaches.

The rhizosphere and root endosphere associated microbial communities of a plant are affected by plant type, plant compartment and environmental factors such as geographic location, soil pH, salinity, moisture content, soil leaching, erosion, and loss of certain nutrients (Pan et al., 2014; Fang et al., 2016; Mukhtar et al., 2018). Microbial diversity associated with the rhizosphere and root endosphere of *Zygophyllum* and *Haloxylon* showed a significant difference as compared to other halophytes, across all geographic sites. The results of sequence analysis of the 16S rRNA gene described that overall maximum microbial diversity identified from the geographic site 2 with more than 98.57% of retrieved sequences assigned to the domain Bacteria and 4.43% sequences related to unclassified microorganisms. Previous studies on Cacti and *Agave* species also reported the similar pattern of microbial diversity (Coleman-Derr et al., 2016; Fonseca-Garcia et al., 2016). At the phylum level, microbial communities identified from the rhizosphere of halophytes showed more diversity as compared to those associated with the root endosphere, across all the three sites. Among the plant species, *Haloxylon* showed overall more microbial diversity as compared to other halophytes, used in this study. Bacterial phyla Actinobacteria, Proteobacteria, Bacteroidetes, Firmicutes, Acidobacteria, Chloroflexi, Planctomycetes, and Fusobacteria were commonly identified from all rhizosphere and root samples. Some previous studies showed that the abundance of certain phyla, such as Actinobacteria, Proteobacteria, Firmicutes, and CRC1 increased in the rhizosphere of desert halophytes such as *Halostachys caspica*, and *Salicornia alterniflora*, with the increase in soil salinity and moisture content (Marasco et al., 2016). Increase in soil salinity also negatively affect the abundance of certain phyla, such as Chloroflexi, Acidobacteria, Nitrospirae, and Synergistetes (Foesel et al., 2014; Yan et al., 2015; Mukhtar et al., 2018).

Overall, Actinobacteria were found to be more abundant in the root endosphere of halophytes than the rhizosphere across all the geographic sites, but within plant species, *Zygophyllum* and *Haloxylon* showed more abundance of Actinobacteria across the geographic site 1 and 3. The results of 16S rRNA based metagenomic analysis showed that bacterial genera *Kocuria, Brevibacterium, Micrococcus, Streptomyces, Solirubrobacter*, and *Nocardia* (Actinobacteria) were identified from the root endosphere of all plants. Previous studies also showed that Actinobacteria are dominant in the salinity and drought affected soils. These bacteria have the potential ability to promote plant growth, to produce a large number of antimicrobial compounds and to degrade a variety of toxic organic compounds from the polluted saline environments (Dupont et al., 2014; Deng et al., 2015; Zhang et al., 2015). Bacterial genera *Asanoa, Actinoplanes, Marmoricola*, and *Microlunatus* were detected only from the rhizosphere of *Aerva* and *Capparis* from the geographic site 1 and 2. These strains have previously been isolated from the rhizosphere of halophytes such as *Phragmites australis, Salsola stocksii*, and *Atriplex amnicola*. These can be used for biofuel production and play an important role in carbon cycling of soil ecosystems (Borruso et al., 2014; Krivushin et al., 2015; Mukhtar et al., 2018).

This study showed that the sequences related to *Proteobacteria* (*Alphaproteobacteria, Betaproteobacteria*, and *Gammaproteobacteria*) were detected from the rhizosphere and root endosphere microbiomes of all the plants but with more abundance in the rhizosphere samples. Bacterial genera *Pseudomonas, Halomonas, Enterobacter, Burkholderia, Azospirillum, Marinobacter*, and *Geobacter* were dominant as compared to other strains in all the rhizosphere samples of all the plants, as compared to root samples across the site 1 and 3 while *Serratia, Azotobacter*, and *Xanthomonas* were identified only from the rhizosphere of *Aerva* and *Capparis*, collected from the geographic site 2. Previous studies also reported the isolation and identification of these Protobacterial genera from a number of saline and arid environments (Jiang et al., 2014; Mukhtar et al., 2018, 2020). These bacteria can be used as biofertilizers, as biocontrol agents and for bioremediation of a variety of hazardous compounds from polluted saline and arid environments (Mukhtar et al., 2017a).

Members of Firmicutes were more abundant in the rhizosphere and root endosphere of *Zygophyllum* and *Haloxylon* as compared to *Capparis* and *Aerva* across all the three sites. As expected from the previous studies, these results also confirmed the correlation of increase in soil salinity and abundance of Firmicutes. *Bacillus, Halobacillus, Virgibacillus, Oceanobacillus, Marinococcus, Planococcus*, and *Exiguobacterium* were dominant genera detected in all the rhizosphere and root endosphere of halophytes. Within plant species, *Haloxylon* showed the maximum diversity of Firmicutes at the genus level between the rhizosphere and root endosphere from the geographic site 2. *Bacillus* strains have plant growth promoting and biocontrol abilities and can be used as potential biofertilizers (Krid et al., 2010; Mukhtar et al., 2017a). *Bacillus* like bacteria isolated and characterized from saline environments have novel halophilic enzymes which can be used for a number of industries and bioremediation of organic pollutants in arid and saline environments (Oren, 2015; Liu et al., 2017; Mukhtar et al., 2017b, 2019).

Dominant genera from the phylum *Bacteroidetes* identified in this study were *Flavobacterium, Salinibacter, Sphingobacterium*, and *Cytophaga.* These genera were detected from the rhizosphere and root endosphere of all the plants across the three geographic sites. *Polaribacter, Chlorobium*, and *Chitinivibrio* were exclusively identified from the rhizosphere and root endosphere of *Aerva* and *Capparis*. Members of Bacteroidetes are usually found to be dominant in the rhizosphere of plants growing under salinity and drought stress conditions (Coleman-Derr et al., 2016; Gibtan et al., 2017). *Gp4, Gp6, Gp7, Gp10*, and *Gp21* (Acidobacteria) were detected in this study. Acidobacteria were more abundant in the rhizosphere as compared to root endosphere of all the halophytes across the site 1 and 3. A number of previous studies also reported the abundance of Acidobacteria in the rhizosphere of halophytes and other hypersaline environments (Zhang et al., 2015; Mukhtar et al., 2016). Sequences related to bacterial phyla Fusobacteria, Gemmatimonadetes, Chloroflexi, Synergistetes, Planctomycetes, Deinococcus-Thermus, Armatimonadetes, and Nitrospirae were identified in the rhizosphere of all the halophytes but they were relatively less abundant across the sites 1 and 3 as compared to the site 2. These phyla have already been detected in various extreme environments such as rhizosphere of halophytes and xerophytes, marine water, rock sediments, anaerobic sludge, and hot spring water (Iverson et al., 2012; Zenga et al., 2014).

## Conclusion

This study described the microbial diversity analysis of the rhizosphere and root endosphere of desert halophytes, collected from the three geographic sites of Cholistan, Pakistan by using 16S rRNA based Illumina sequencing. The present study reveals that plant species, plant compartments and a change in soil physicochemical characteristics affect the composition of rhizosphere and root endophytic microbial communities. Phylogenetic profiling showed that bacterial phyla Actinobacteria, Proteobacteria, Bacteroidetes, Firmicutes, and Fusobacteria were commonly identified from all the rhizosphere and root samples with significant variations between the rhizosphere and root endosphere, plant species across the three sites. Microbial diversity associated with the rhizosphere and root endosphere of *Haloxylon* collected from the geographic site 2 showed the greatest variations at the phylum and genus level. From the rhizosphere and the roots of each plant, certain bacterial genera were more dominant. More than 80% bacterial genera identified in this study have the potential ability to promote plant growth under salinity and drought stress environments and could be used as potential candidates for biofertilizers in salt affected agricultural lands.

## Data Availability Statement

The datasets presented in this study can be found in online repositories. The names of the repository/repositories and accession number(s) can be found below: https://www.ncbi.nlm.nih.gov/, SRR9588854-SRR9588861.

## Author Contributions

SMu: conducted the experiment, analyzed the data, and prepared the manuscript. SMe: guided in experiment plan and edited the manuscript. KM: supervised the research and edited the manuscript. All authors contributed to the article and approved the submitted version.

## Conflict of Interest

The authors declare that the research was conducted in the absence of any commercial or financial relationships that could be construed as a potential conflict of interest.
