# Supplementary material for: Comparative Study of the Rhizosphere and Root Endosphere Microbiomes of Cholistan Desert Plants
Source: Front Microbiol. 2021 Mar 26;12:618742. doi: 10.3389/fmicb.2021.618742 (PMC8032897; doi:10.3389/fmicb.2021.618742)
Supplement: Supplementary file 1 [file Data_Sheet_1.docx]

**Supplementary information**

**Comparison of the rhizosphere and root endosphere microbiomes of Cholistan desert plants across the three sampling sites**

**Salma Mukhtar^1,2^, Samina Mehnaz^1^ and Kauser Abdulla Malik^1^***

**^1^School of Life Sciences, Forman Christian College (A Chartered University), Ferozepur Road, Lahore 54600,** Pakistan.

^2^School of Biological Sciences, University of the Punjab, Lahore, Pakistan.


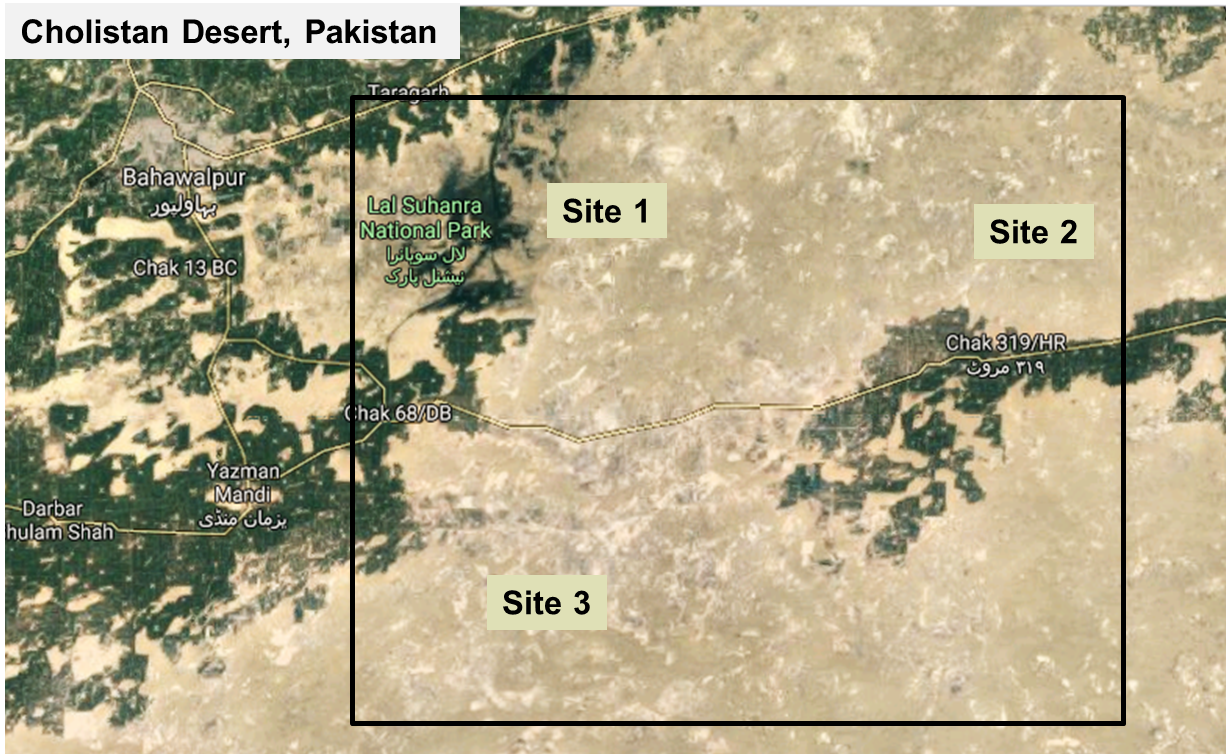


**Figure S1.** Geological information of sampling area, Cholistan Desert, Pakistan

**
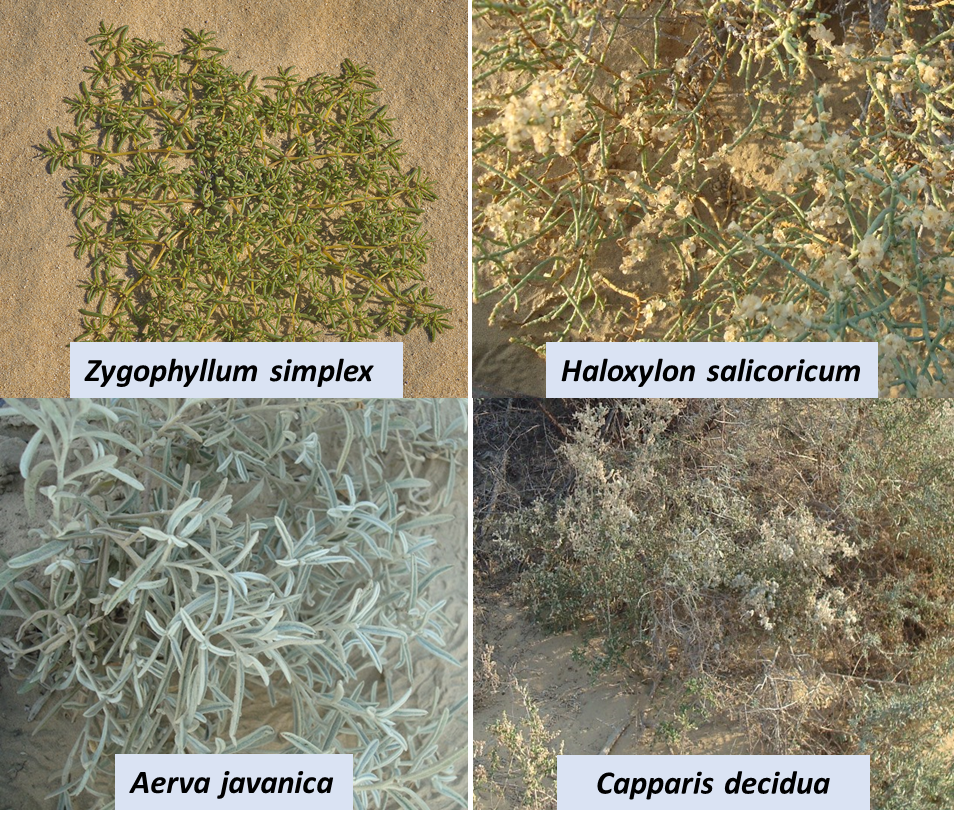
**

**Figure S2.** Sampling of rhizospheric soil and roots of xerophytes *Zygophyllum simplex, Haloxylon salicoricum, Aerva javanica* and *Capparis decidua* from four sites of Cholistan desert, Pakistan


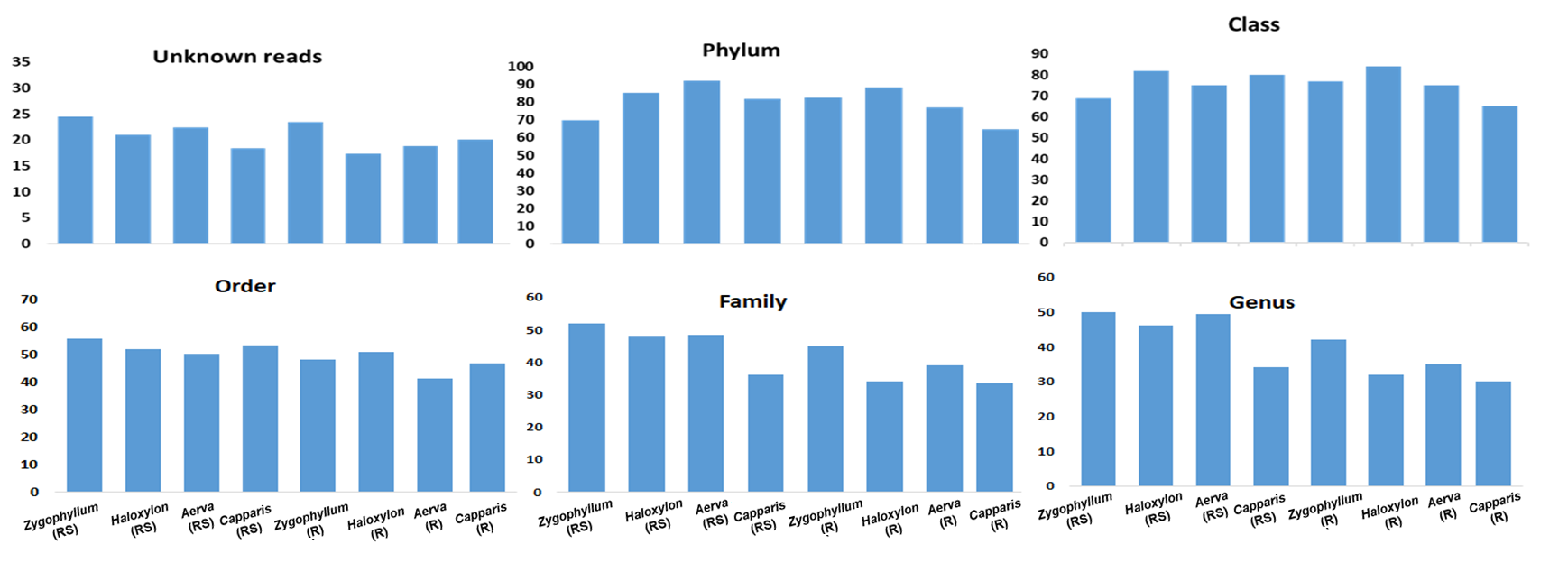


**Figure S3.** Number of sequences assigned by RDP multi-classifier for each soil sample at each taxonomic level (phylum, class, order, family and genus) for rhizospheric soils and root endosphere samples collected from site 1


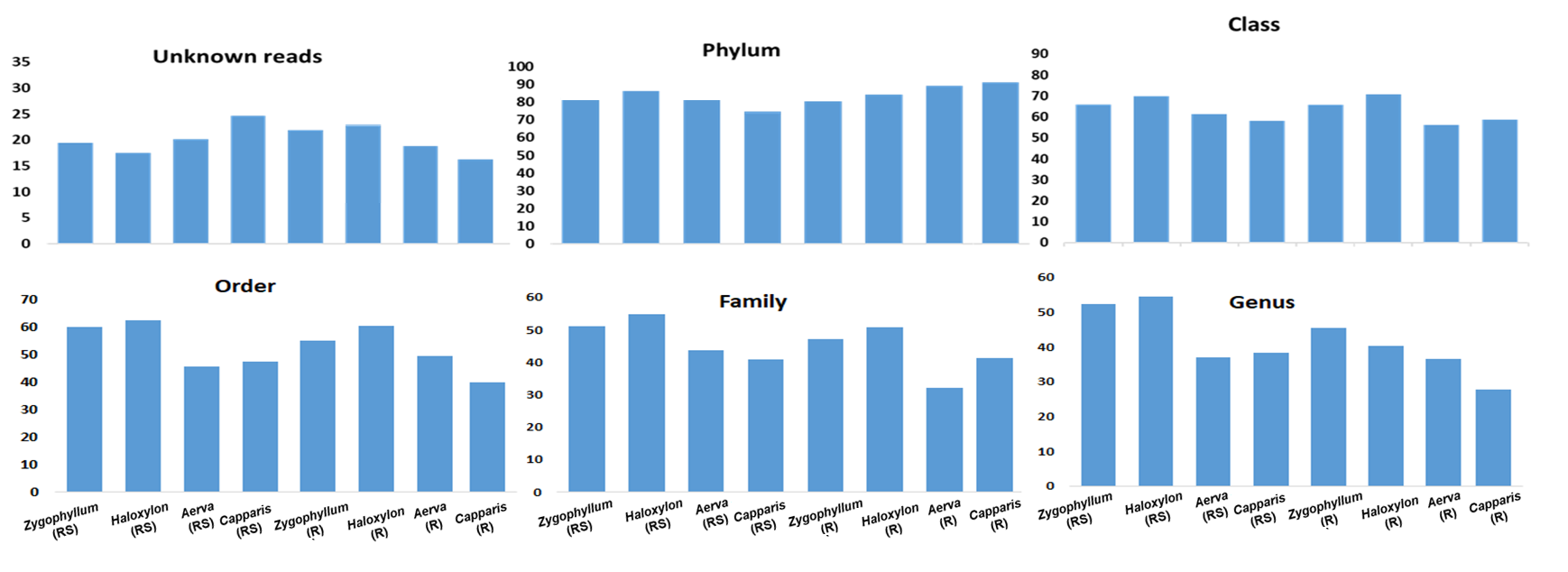


**Figure S4.** Number of sequences assigned by RDP multi-classifier for each soil sample at each taxonomic level (phylum, class, order, family and genus) for rhizospheric soils and root endosphere samples collected from site 2

**
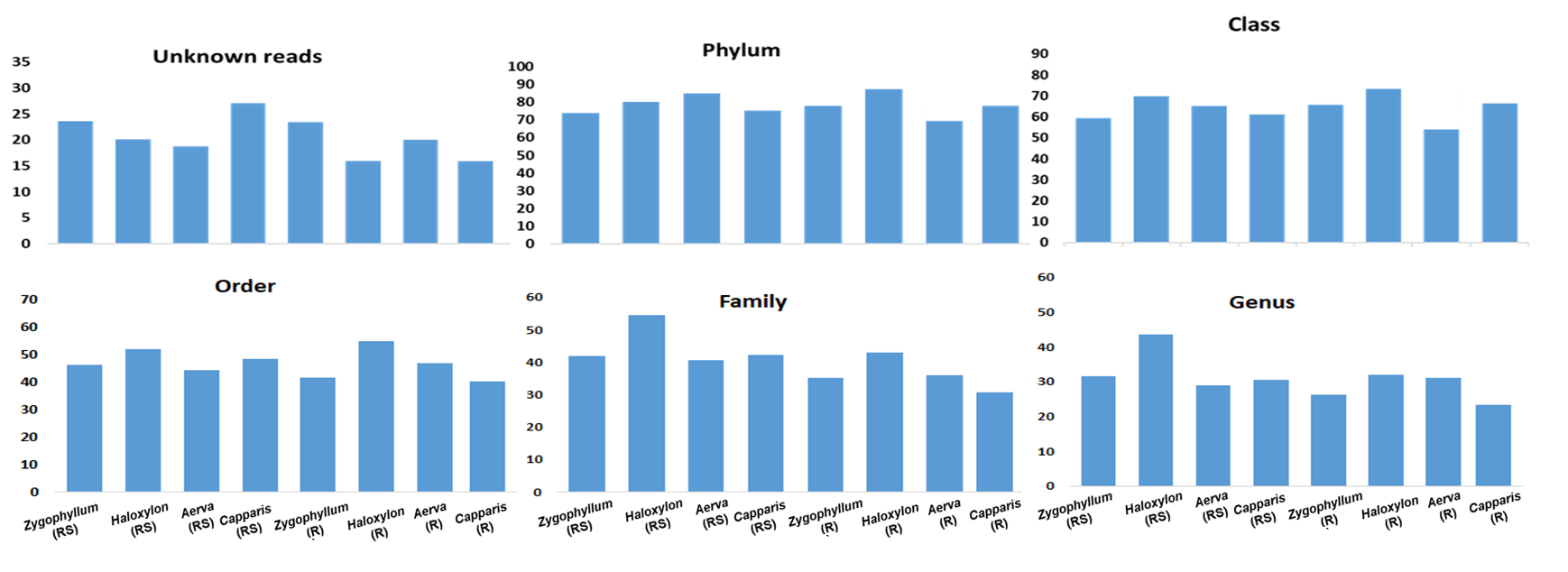
**

**Figure S5.** Number of sequences assigned by RDP multi-classifier for each soil sample at each taxonomic level (phylum, class, order, family and genus) for rhizospheric soils and root endosphere samples collected from site 3

**
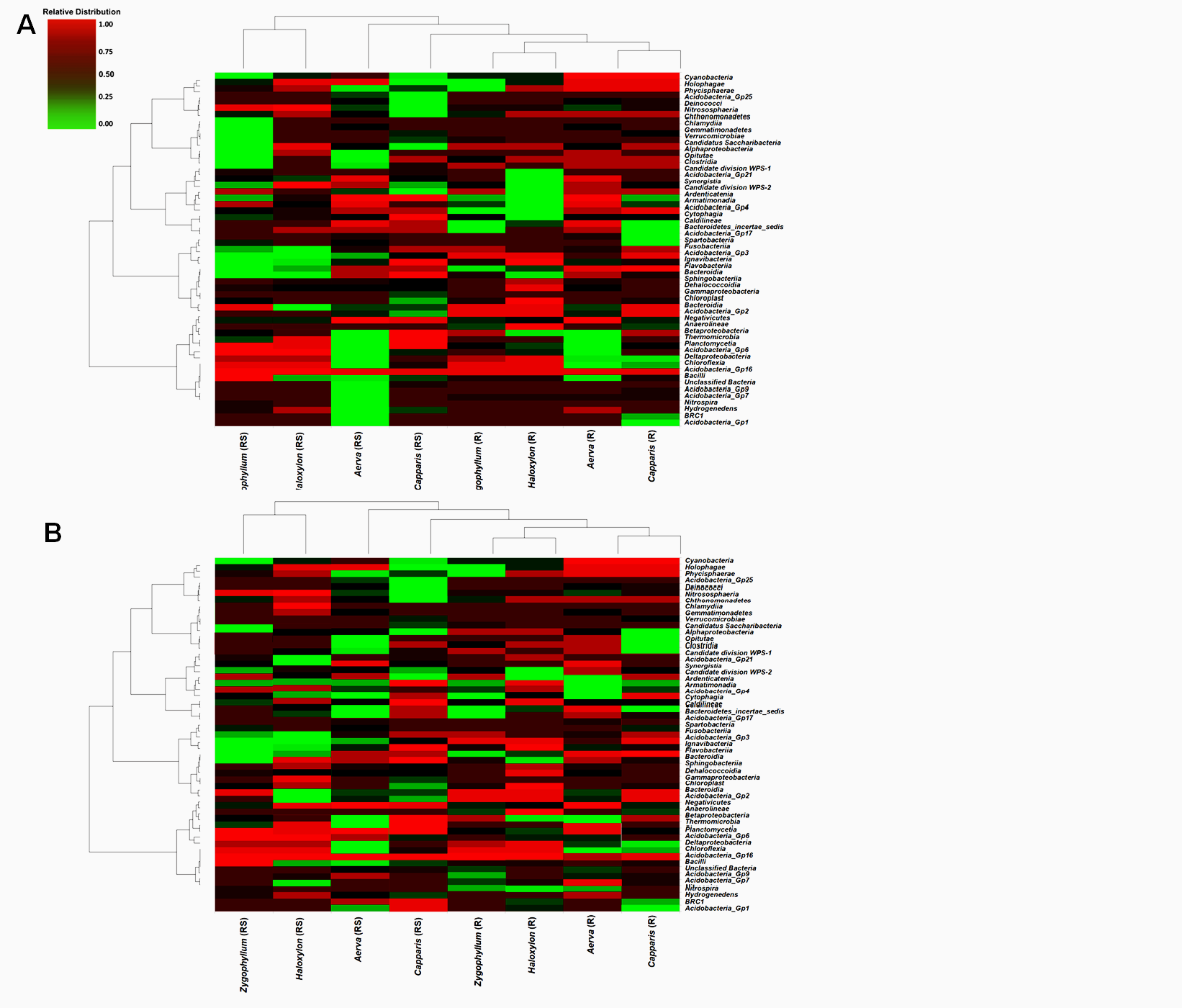
**

**
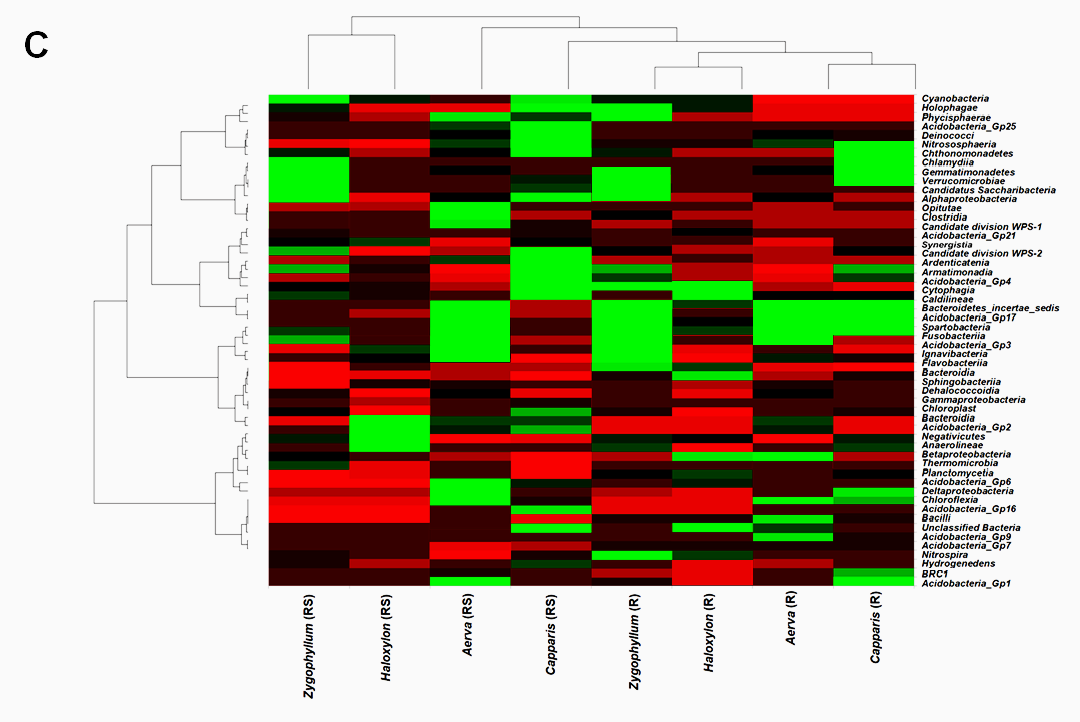
**

**Figure S6** The heatmap reports the normalized values of the taxonomic assignments at class level.

Each value has been normalized following this criterion:

where X_ij_ is the occurrence of the classes ‘j’ in the soil/root sample ‘i’ and N is the number of soil and root samples (in this study 24) collected from three sites; **(A)** site 1, **(B)** site 2 and **(C)** site 3. Using this transformation, sequences assigned to each class can be compared in all soil and root samples independently from its order of magnitude.

**
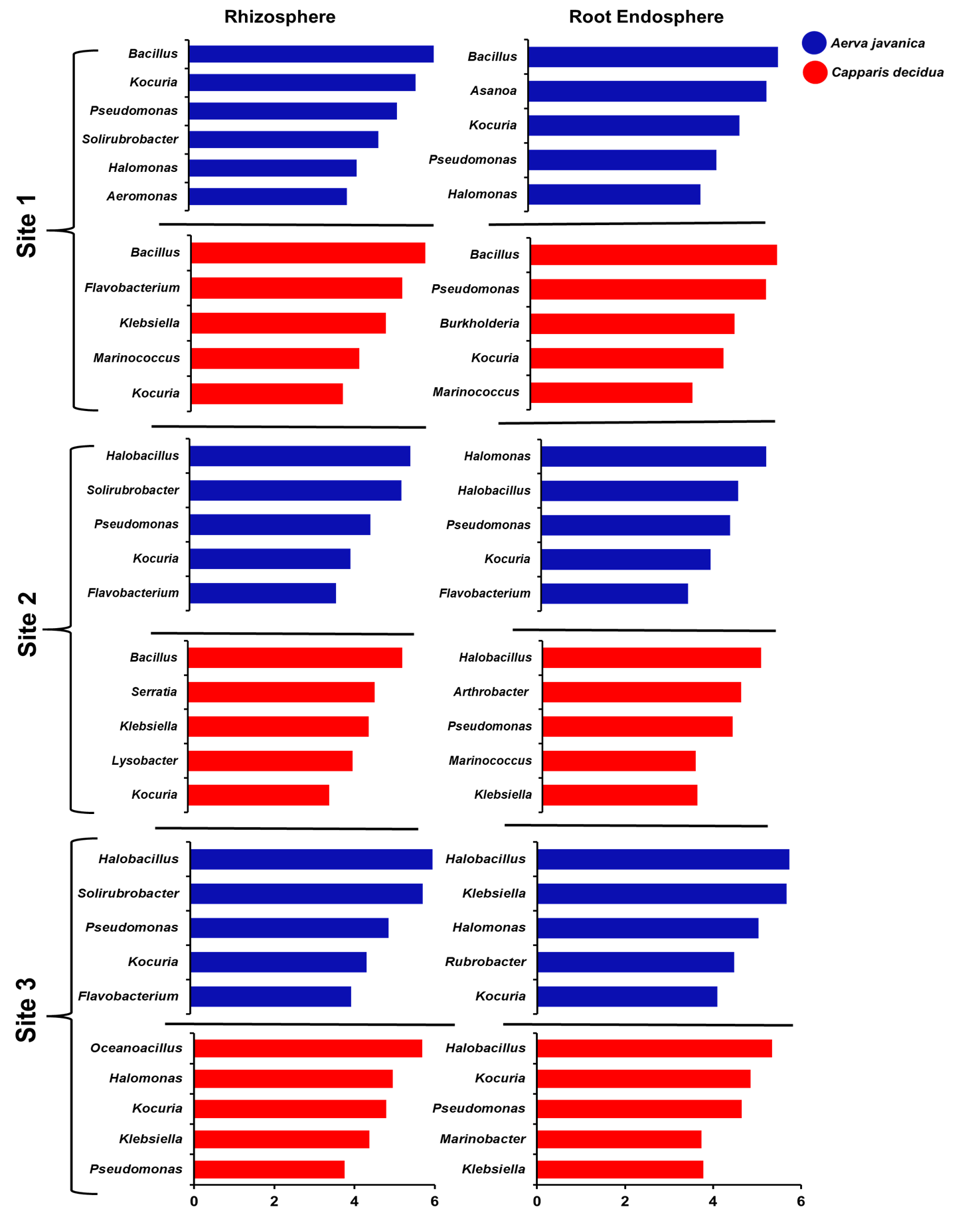
**

**Figure S7.** The results of LEfSe analysis showed the identified bacterial genera that were significantly abundant in the rhizosphere and root endosphere of halophytes. Variations in the bacterial genera detected across the rhizosphere and the root endosphere of *Aerva* and *Capparis* from 3 sampling sites. Bacterial genera with the LDA score of more than 3.5 are shown.

**Table S1.** Primers and barcode sequences used for targeting bacterial communities from the rhizosphere and root endosphere of desert halophytes

| **Plant** | **Sample ID** | **Barcode sequence** | **Primer sequence (5΄-3΄)** |
| --- | --- | --- | --- |
| *Zygophyllum* rhizosphere | ZM-RS | AGGAGTGCGT | Bakt_341F: CCTACGGGNGGCWGCAG |
| *Zygophyllum* root endosphere | ZM-RR | ACGTGTCGAA | Bakt_805R: GACTACHVGGGTATCTAATCC |
| *Haloxylon* rhizosphere | HAL-RS | AGAAGCACTC |  |
| *Haloxylon* root endosphere | HAL-RR | AGCAATGTAG |  |
| *Aerva* rhizosphere | AV-RS | ATCAGTACAC |  |
| *Aerva* root endosphere | AV-RR | ATTACGCGAG |  |
| *Capparis* rhizosphere | OP-RS | CCTGTCTGTA |  |
| *Capparis* root endosphere | OP-RR | CAGCGCTGTC |  |

**Table S2.** Number of effective reads obtained from the rhizospheric soils and root samples of halophytes, collected from three sites

| **Sampling site** | **Sample type** | **Total reads before removal of mitochondrial and plastid contaminants** | | | | **Total reads after removal of mitochondrial and plastid contaminants** | | | |
| --- | --- | --- | --- | --- | --- | --- | --- | --- | --- |
|  |  | ***Zygophyllum*** | ***Haloxylon*** | ***Aerva*** | ***Capparis*** | ***Zygophyllum*** | ***Haloxylon*** | ***Aerva*** | ***Capparis*** |
| **1** | Rhizosphere | 60985 | 65823 | 43952 | 52467 | 55674 | 60842 | 41359 | 47623 |
|  | Root endosphere | 51653 | 63628 | 43743 | 45597 | 43287 | 49527 | 35982 | 37541 |
| **2** | Rhizosphere | 65954 | 69327 | 47632 | 45674 | 61131 | 63943 | 43691 | 41379 |
|  | Root endosphere | 57861 | 61423 | 49627 | 37659 | 49657 | 54621 | 38924 | 33591 |
| **3** | Rhizosphere | 63459 | 75548 | 51598 | 55324 | 57587 | 69584 | 45953 | 49526 |
|  | Root endosphere | 56843 | 69547 | 50347 | 59874 | 45965 | 57635 | 39745 | 43531 |

**Table S3.** Mean estimated OTUs with standard errors associated with the rhizosphere and root endosphere of desert halophytes

| **Sampling site** | **Sample type** | **Plant species** | | | |
| --- | --- | --- | --- | --- | --- |
|  |  | ***Zygophyllum ^c^*** | ***Haloxylon ^d^*** | ***Aerva ^e^*** | ***Capparis ^f^*** |
| **1** | Rhizosphere ^a^ | 140.56 ± 16.254 | 244.85 ± 21.452 | 198.15 ± 12.147 | 175.27 ± 11.856 |
|  | Root endosphere ^b^ | 122.23 ± 13.530 | 209.47 ± 19.131 | 175.76 ± 10.834 | 140.59 ± 10.742 |
| **2** | Rhizosphere ^a^ | 170.48 ± 20.032 | 277.97 ± 13.943 | 169.85 ± 13.752 | 169.52 ± 9.641 |
|  | Root endosphere ^b^ | 130.52 ± 11.512 | 230.51 ± 21.331 | 146.46 ± 11.853 | 134.75 ± 12.343 |
| **3** | Rhizosphere ^a^ | 149.06 ± 14.641 | 212.17 ± 23.974 | 178.31 ± 15.104 | 168.21 ± 10.542 |
|  | Root endosphere ^b^ | 130.53 ± 9.304 | 220.25 ± 17.091 | 135.25 ± 9.512 | 141.17 ± 9.671 |

Note:

1. Statistically significant difference among rhizosphere samples from all three sites after Kruskal-Wallis test for bacterial species (*H*2, 41 = 21.942, *P*=0.0313).
2. Statistically significant difference among root endosphere samples from all three sites after Kruskal-Wallis test for bacterial species (*H*2, 41 = 13.129, *P*=0.0241)
3. Statistically significant difference between rhizosphere and root endosphere samples of *Zygophyllum* after Kruskal-Wallis test for bacterial species (*H*2, 42 = 29.691, *P*=0.0013)
4. Statistically significant difference between rhizosphere and root endosphere samples of *Haloxylon* after Kruskal-Wallis test for bacterial species (*H*2, 42 = 35.547, *P*=0.007)
5. Statistically significant difference between rhizosphere and root endosphere samples of *Aerva* after Kruskal-Wallis test for bacterial species (*H*2, 42 = 27.455, *P*=0.00011)
6. Statistically significant difference between rhizosphere and root endosphere samples of *Capparis* after Kruskal-Wallis test for bacterial species (*H*2, 42 = 39.543, *P*=0.0015)

**Table S4.** Mean estimated Shannon diversity with standard errors associated with the rhizosphere and root endosphere of desert halophytes

| **Sampling site** | **Sample type** | **Plant species** | | | |
| --- | --- | --- | --- | --- | --- |
|  |  | ***Zygophyllum ^c^*** | ***Haloxylon ^d^*** | ***Aerva ^e^*** | ***Capparis ^f^*** |
| **1** | Rhizosphere ^a^ | 4.86 ± 0.341 | 5.83 ± 1.303 | 4.27 ± 0.341 | 4.58 ± 0.835 |
|  | Root endosphere ^b^ | 3.63 ± 0.511 | 4.92 ± 1.512 | 3.74 ± 0.218 | 3.15 ± 0.242 |
| **2** | Rhizosphere ^a^ | 5.43 ± 1.011 | 6.23 ± 1.213 | 4.05 ± 0.733 | 4.32 ± 0.431 |
|  | Root endosphere ^b^ | 4.77 ± 0.952 | 5.35 ± 0.974 | 4.46 ± 0.542 | 3.31 ± 0.355 |
| **3** | Rhizosphere ^a^ | 4.21 ± 1.041 | 5.29 ± 0.753 | 4.17 ± 0.764 | 4.44 ± 0.529 |
|  | Root endosphere ^b^ | 3.53 ± 0.540 | 4.15 ± 0.356 | 3.75 ± 0.553 | 3.33 ± 0.471 |

Note:

1. Statistically significant difference among rhizosphere samples from all three sites after Kruskal-Wallis test for bacterial species (*H*2, 41 = 7.368, *P*=0.0414).
2. Statistically significant difference among root endosphere samples from all three sites after Kruskal-Wallis test for bacterial species (*H*2, 41 = 19.959, *P*=0.01487)
3. Statistically significant difference between rhizosphere and root endosphere samples of *Zygophyllum* after Kruskal-Wallis test for bacterial species (*H*2, 42 = 1.427, *P*=0.0005)
4. Statistically significant difference between rhizosphere and root endosphere samples of *Haloxylon* after Kruskal-Wallis test for bacterial species (*H*2, 42 = 27.161, *P*=0.0017)
5. Statistically significant difference between rhizosphere and root endosphere samples of *Aerva* after Kruskal-Wallis test for bacterial species (*H*2, 42 = 16.524, *P*=0.0007)
6. Statistically significant difference between rhizosphere and root endosphere samples of *Capparis* after Kruskal-Wallis test for bacterial species (*H*2, 42 = 19.787, *P*=0.0003)

**Table S5.** Average relative abundance of top 5 OTUs with an abundance >1% at family and genus level from the rhizosphere and root endosphere samples of desert halophytes collected from site 1

| **Plant** | **Sample type** | Family | % | Genus | % |
| --- | --- | --- | --- | --- | --- |
| ***Zygophyllum*** | Rhizosphere | Bacillaceae | 12.11 | *Bacillus* | 5.98 |
|  |  | Micrococcaceae | 11.51 | *Kocuria* | 5.37 |
|  |  | Pseudomonadaceae | 8.27 | *Pseudomonas* | 4.75 |
|  |  | Microbacteriaceae | 6.45 | *Microbacterium* | 3.77 |
|  |  | Aeromonadaceae | 5.65 | *Aeromonas* | 3.17 |
|  | Root endosphere | Bacillaceae | 11.53 | *Virgibacillus* | 8.49 |
|  |  | Micrococcaceae | 10.27 | *Kocuria* | 6.23 |
|  |  | Halomonadaceae | 8.14 | *Halomonas* | 5.25 |
|  |  | Rubrobacteraceae | 6.69 | *Rubrobacter* | 4.83 |
|  |  | Pseudomonadaceae | 5.19 | *Pseudomonas* | 3.11 |
| ***Haloxylon*** | Rhizosphere | Bacillaceae | 13.57 | *Bacillus* | 12.95 |
|  |  | Alteromonadaceae | 4.63 | *Marinobacter* | 10.67 |
|  |  | Flavobacteriaceae | 7.77 | *Flavobacterium* | 7.85 |
|  |  | Micrococcaceae | 10.27 | *Kocuria* | 4.23 |
|  |  | Rubrobacteraceae | 21.52 | *Rubrobacter* | 1.57 |
|  | Root endosphere | Bacillaceae | 10.77 | *Bacillus* | 8.15 |
|  |  | Micrococcaceae | 8.51 | *Kocuria* | 6.67 |
|  |  | Conexibacteraceae | 7.24 | *Conexibacter* | 5.89 |
|  |  | Planococcusaceae | 4.75 | *Planococcus* | 3.23 |
|  |  | Alteromonadaceae | 3.61 | *Marinobacter* | 2.19 |
| ***Aerva*** | Rhizosphere | Bacillaceae | 11.35 | *Bacillus* | 8.71 |
|  |  | Micrococcaceae | 9.11 | *Kocuria* | 7.17 |
|  |  | Pseudomonadaceae | 7.27 | *Pseudomonas* | 5.57 |
|  |  | Flavobacteriaceae | 5.35 | *Flavobacterium* | 3.81 |
|  |  | Halomonadaceae | 3.83 | *Halomonas* | 2.23 |
|  | Root endosphere | Bacillaceae | 13.17 | *Bacillus* | 6.79 |
|  |  | Micromonosporaceae | 7.87 | *Asanoa* | 5.91 |
|  |  | Micrococcaceae | 7.41 | *Kocuria* | 4.27 |
|  |  | Pseudomonadaceae | 4.63 | *Pseudomonas* | 3.33 |
|  |  | Halomonadaceae | 3.54 | *Halomonas* | 2.15 |
| ***Capparis*** | Rhizosphere | Bacillaceae | 13.23 | *Bacillus* | 5.91 |
|  |  | Flavobacteriaceae | 8.37 | *Flavobacterium* | 5.25 |
|  |  | Enterobacteriaceae | 7.19 | *Klebsiella* | 4.13 |
|  |  | Micromonosporaceae | 4.77 | *Asanoa* | 2.87 |
|  |  | Micrococcaceae | 3.55 | *Kocuria* | 2.07 |
|  | Root endosphere | Bacillaceae | 11.57 | *Bacillus* | 6.21 |
|  |  | Pseudomonadaceae | 7.33 | *Pseudomonas* | 4.63 |
|  |  | Flavobacteriaceae | 5.23 | *Flavobacterium* | 3.61 |
|  |  | Micrococcaceae | 4.19 | *Kocuria* | 3.12 |
|  |  | Halomonadaceae | 3.11 | *Halomonas* | 1.75 |

**Table S6.** Average relative abundance of top 5 OTUs with an abundance >1% at family and genus level from the rhizosphere and root endosphere samples of desert halophytes collected from site 2

| **Plant** | **Sample type** | Family | % | Genus | % |
| --- | --- | --- | --- | --- | --- |
| ***Zygophyllum*** | Rhizosphere | Bacillaceae | 13.17 | *Halobacillus* | 9.79 |
|  |  | Pseudonocardiaceae | 11.37 | *Actinomycetospora* | 7.49 |
|  |  | Pseudomonadaceae | 8.45 | *Pseudomonas* | 5.857 |
|  |  | Micrococcaceae | 7.13 | *Arthrobacter* | 3.51 |
|  |  | Microbacteriaceae | 5.41 | *Microbacterium* | 2.31 |
|  | Root endosphere | Bacillaceae | 11.29 | *Bacillus* | 5.79 |
|  |  | Halomonadaceae | 8.47 | *Halomonas* | 5.63 |
|  |  | Pseudomonadaceae | 6.15 | *Pseudomonas* | 3.27 |
|  |  | Micrococcaceae | 5.21 | *Arthrobacter* | 3.33 |
|  |  | Microbacteriaceae | 3.49 | *Microbacterium* | 2.29 |
| ***Haloxylon*** | Rhizosphere | Bacillaceae | 12.81 | *Halobacillus* | 9.27 |
|  |  | Micrococcaceae | 11.11 | *Kocuria* | 7.31 |
|  |  | Aeromonadaceae | 9.77 | *Aeromonas* | 5.49 |
|  |  | Flavobacteriaceae | 7.24 | *Flavobacterium* | 4.45 |
|  |  | Alteromonadaceae | 4.35 | *Marinobacter* | 2.51 |
|  | Root endosphere | Bacillaceae | 13.14 | *Bacillus* | 8.42 |
|  |  | Enterobacteriaceae | 10.65 | *Enterobacter* | 6.37 |
|  |  | Flavobacteriaceae | 8.15 | *Flavobacterium* | 4.87 |
|  |  | Micrococcaceae | 6.51 | *Kocuria* | 3.73 |
|  |  | Halomonadaceae | 4.28 | *Halomonas* | 2.19 |
| ***Aerva*** | Rhizosphere  Flavobacterium | Bacillaceae | 13.19 | *Halobacillus* | 9.35 |
|  |  | Burkholderiaceae | 8.11 | *Burkholderia* | 7.71 |
|  |  | Micrococcaceae | 7.13 | *Kocuria* | 5.23 |
|  |  | Flavobacteriaceae | 4.54 | *Flavobacterium* | 3.47 |
|  |  | Pseudomonadaceae | 3.17 | *Pseudomonas* | 2.39 |
|  | Root endosphere | Halomonadaceae | 11.29 | *Halomonas* | 7.31 |
|  |  | Bacillaceae | 9.41 | *Halobacillus* | 6.55 |
|  |  | Pseudomonadaceae | 5.83 | *Pseudomonas* | 4.33 |
|  |  | Micrococcaceae | 4.91 | *Kocuria* | 3.24 |
|  |  | Flavobacteriaceae | 3.13 | *Flavobacterium* | 1.77 |
| ***Capparis*** | Rhizosphere | Bacillaceae | 11.42 | *Bacillus* | 5.73 |
|  |  | Flavobacteriaceae | 7.53 | *Serratia* | 5.07 |
|  |  | Enterobacteriaceae | 5.59 | *Klebsiella* | 4.25 |
|  |  | Flavobacteriaceae | 4.21 | *Polaribacter* | 2.51 |
|  |  | Micrococcaceae | 2.77 | *Kocuria* | 1.65 |
|  | Root endosphere | Bacillaceae | 12.19 | *Halobacillus* | 7.27 |
|  |  | Micrococcaceae | 7.57 | *Arthrobacter* | 5.63 |
|  |  | Pseudomonadaceae | 5.51 | *Pseudomonas* | 3.15 |
|  |  | Flavobacteriaceae | 3.69 | *Polaribacter* | 2.27 |
|  |  | Halomonadaceae | 3.01 | *Halomonas* | 1.83 |

| **Plant** | **Sample type** | Family | % | Genus | % |
| --- | --- | --- | --- | --- | --- |
| ***Zygophyllum*** | Rhizosphere | Bacillaceae | 12.41 | *Bacillus* | 8.71 |
|  |  | Pseudomonadaceae | 11.24 | *Pseudomonas* | 8.24 |
|  |  | Alteromonadaceae | 8.15 | *Marinobacter* | 6.57 |
|  |  | Micrococcaceae | 7.63 | *Arthrobacter* | 4.47 |
|  |  | Solirubrobacteraceae | 5.11 | *Solirubrobacter* | 3.17 |
|  | Root endosphere | Bacillaceae | 11.71 | *Halobacillus* | 7.51 |
|  |  | Halomonadaceae | 10.81 | *Halomonas* | 6.13 |
|  |  | Aeromonadaceae | 7.67 | *Aeromonas* | 4.55 |
|  |  | Micrococcaceae | 5.79 | *Kocuria* | 3.63 |
|  |  | Alteromonadaceae | 4.63 | *Marinobacter* | 2.55 |
| ***Haloxylon*** | Rhizosphere | Bacillaceae | 12.35 | *Halobacillus* | 8.64 |
|  |  | Pseudomonadaceae | 9.21 | *Pseudomonas* | 7.11 |
|  |  | Halomonadaceae | 7.37 | *Halomonas* | 4.41 |
|  |  | Micrococcaceae | 5.23 | *Kocuria* | 3.11 |
|  |  | Enterobacteriaceae | 3.52 | *Citrobacter* | 2.36 |
|  | Root endosphere | Bacillaceae | 12.69 | *Bacillus* | 7.35 |
|  |  | Micrococcaceae | 8.11 | *Kocuria* | 6.49 |
|  |  | Enterobacteriaceae | 6.51 | *Enterobacter* | 3.67 |
|  |  | Halomonadaceae | 4.59 | *Halomonas* | 3.21 |
|  |  | Flavobacteriaceae | 3.44 | *Flavobacterium* | 2.15 |
| ***Aerva*** | Rhizosphere | Bacillaceae | 12.15 | *Halobacillus* | 7.45 |
|  |  | Solirubrobacteraceae | 8.51 | *Solirubrobacter* | 5.61 |
|  |  | Pseudomonadaceae | 6.27 | *Pseudomonas* | 4.43 |
|  |  | Micrococcaceae | 5.34 | *Kocuria* | 4.15 |
|  |  | Flavobacteriaceae | 3.59 | *Flavobacterium* | 2.64 |
|  | Root endosphere | Bacillaceae | 12.57 | *Halobacillus* | 6.93 |
|  |  | Enterobacteriaceae | 7.73 | *Klebsiella* | 5.11 |
|  |  | Halomonadaceae | 6.15 | *Halomonas* | 3.85 |
|  |  | Rubrobacteraceae | 4.73 | *Rubrobacter* | 3.38 |
|  |  | Micrococcaceae | 3.49 | *Kocuria* | 2.37 |
| ***Capparis*** | Rhizosphere | Bacillaceae | 11.32 | *Oceanoacillus* | 5.59 |
|  |  | Halomonadaceae | 8.47 | *Halomonas* | 4.35 |
|  |  | Micrococcaceae | 6.57 | *Kocuria* | 3.69 |
|  |  | Flavobacteriaceae | 4.59 | *Flavobacterium* | 2.74 |
|  |  | Pseudomonadaceae | 3.23 | *Pseudomonas* | 2.05 |
|  | Root endosphere | Bacillaceae | 10.15 | *Halobacillus* | 6.31 |
|  |  | Micrococcaceae | 7.57 | *Kocuria* | 5.29 |
|  |  | Pseudomonadaceae | 6.17 | *Pseudomonas* | 3.37 |
|  |  | Alteromonadaceae | 3.53 | *Marinobacter* | 2.67 |
|  |  | Flavobacteriaceae | 2.75 | *Polaribacter* | 1.55 |

**Table S7.** Average relative abundance of top 5 OTUs with an abundance >1% at family and genus level from the rhizosphere and root endosphere samples of desert halophytes collected from site 3
